# Recent Developments of Versatile Photoinitiating Systems for Cationic Ring Opening Polymerization Operating at Any Wavelengths and under Low Light Intensity Sources

**DOI:** 10.3390/molecules20047201

**Published:** 2015-04-20

**Authors:** Jacques Lalevée, Haifaa Mokbel, Jean-Pierre Fouassier

**Affiliations:** Institut de Science des Matériaux de Mulhouse IS2M, UMR CNRS 7361, UHA, 15, rue Jean Starcky, 68057 Mulhouse Cedex, France; E-Mails: haifaa.mokbel@uha.fr (H.M.); jp.fouassier@uha.fr (J.-P.F.)

**Keywords:** photoinitiator, cationic polymerization, free radical promoted cationic polymerization, visible light, LED, laser diode

## Abstract

Photoinitiators (PI) or photoinitiating systems (PIS) usable in light induced cationic polymerization (CP) and free radical promoted cationic polymerization (FRPCP) reactions (more specifically for cationic ring opening polymerization (ROP)) together with the involved mechanisms are briefly reviewed. The recent developments of novel two- and three-component PISs for CP and FRPCP upon exposure to low intensity blue to red lights is emphasized in details. Examples of such reactions under various experimental conditions are provided.

## 1. Introduction

Ring opening polymerization (ROP) reactions are very well known in macromolecular science (see e.g., in [1,2]). Thermally initiated ionic and coordination ROP as well as controlled ROP have been recently reviewed and detailed in [3]. Cationic polymerization (CP) reactions (of e.g., divinylethers) and the particular cationic ROP of cyclic monomers (e.g., epoxides, oxiranes such as cyclohexene oxide) can also be initiated by light (see, e.g., in books [4,5,6,7,8,9,10,11,12,13,14,15,16] and in review papers [17,18,19,20,21,22,23,24,25,26,27,28,29,30,31,32]).

The applications of light induced CP are extensively developed in the Radiation Curing (RC) area (e.g., in coatings). Applications are also noted, e.g., in printing inks, microelectronics or manufacture of optical elements. On industrial grounds, the benefits of the RC technology are, e.g., use of non-polluting and solvent-free formulations (almost no Volatile Organic Compounds (VOC)), low energy requirements, low temperature treatments (suitable for heat sensitive substrates), rapid through-cure, small space requirements, and low costs. Cationic photopolymerizations are, however, less encountered than free radical photopolymerization reactions. Indeed, although they exhibit some decisive advantages (e.g., no oxygen inhibition, important dark post-effect, less shrinkage problems in the cured material), they present several drawbacks (e.g., moisture and water sensitivity, very limited choice of monomer formulations, slower rates of polymerization for the epoxides, higher costs for monomers). The range of monomers and oligomers available for ROP has been, however, largely expanded. Epoxides are widely used and give coatings with high thermal capability, excellent adhesion, good chemical resistance and environmentally friendly characteristics. Cyclic ethers such as oxetanes are alternatives to epoxides for getting fast curing speeds in industrial lines. Recent developments include, e.g., cationic monomers possessing readily abstractable hydrogen atoms, mono and bifunctional epoxides, hybrid monomers such as epoxide-vinyl ethers (or -propenyl ethers, -acetals), spiro orthocarbonates, epoxy modified silicone monomers, renewable monomers (e.g., epoxidized sunflower, soybean oil, linseed oil, vernonia oil, castor oil; limonene dioxide LDO, epoxidized natural rubbers).

**Scheme 1:**
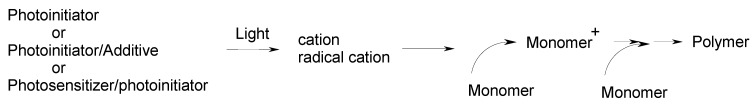
General scheme for cationic photoinitiator.

CP reactions require the presence of a photoinitiator PI or a photoinitiating system PIS (consisting in a photosensitizer PS/PI couple or a PI/additive(s) system) that can absorb the light and generate cations or radical cations being able to initiate CP (Scheme 1). The absorption properties, the excited state processes, the easiness of production of the initiating species as well as their reactivity towards the addition reaction to the cationic monomer ring govern the overall efficiency of the polymerization reaction.

Conventional polychromatic UV and visible light sources (Hg lamps, Xe-Hg lamps, Xe lamps, doped Hg lamps) are usually employed. The recent design of high intensity quasi-monochromatic LED or monochromatic laser diode arrays as well as the use of sunlight (in sunlight-assisted processes: no irradiation device, possibility of curing large dimension pieces or surfaces) or low intensity household devices delivering visible lights (no UV rays, no Hg lamps, no ozone release) allow new opportunities of applications.

**Scheme 2:**
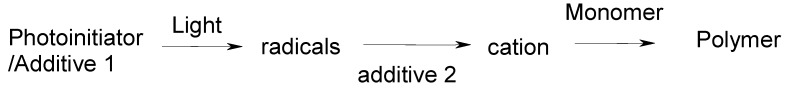
Principle for the free radical promoted cationic polymerization.

Both the relatively small number of originally available cationic PIs and the difficulty to find efficient photosensitizers (PSs) have forced CP to mostly operate under UV lights for a long time, even though some interesting novel PIs or PS/PI couples already ensured long wavelength sensitivity (see below). Today, the development of novel free radical promoted cationic polymerization (FRPCP) reactions (Scheme 2) using well adapted photoinitiating systems (PISs) (in order to decrease the oxygen inhibition due to the radicals) has noticeably enhanced the possibilities and allowed a photosensitivity from the violet to the red (see below).

In the present paper, we will briefly present the PIs and PISs that have been developed for CP and FRPCP reactions, more specifically for the cationic ROP of epoxides. The involved mechanisms will also be reviewed. As many review papers and books on the currently existing PIs and PISs already appeared (see above), we will focus the attention of the reader on a recently newly proposed strategy for the design of original two- and three-component PISs for CP and FRPCP upon exposure to low intensity blue to red lights. Several examples will be provided.

## 2. Backgrounds: Photoinitiation of Cationic ROP

Iodonium (Iod) and sulfonium (Sulf) salts, originally proposed in the 1970s [33,34] (see also reviews in [5,29]) and extensively studied [35,36,37,38,39,40,41,42] are the most widely used PIs for the CP of a large variety of monomers such as epoxides. In the near UV, the simplest Iod (diphenyl iodonium salt Ph_2_I^+^) exhibits a less intense absorption (located at ~230 nm) than the corresponding triphenyl sulfonium salt Ph_3_S^+^ (absorption at ~260 nm).

**Scheme 3:**
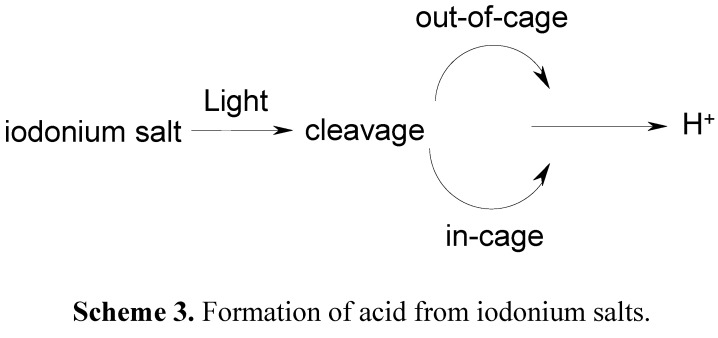
Formation of acid from iodonium salts.

The photodecomposition process is well established and involves a primary heterolytic or/and homolytic cleavage of the C-I or C-S bond. A phenyliodide/phenyl cation or a phenyliodinium cation/phenyl radical pair is thus produced in irradiated Iod. In Sulf, a heterolytic cleavage also occurs. Further out-of-cage processes (due to the presence of abstractable hydrogen atom in the medium) and in-cage processes arise; in any case, a strong Brönsted acid is generated and can initiate ROP (Scheme 3).

**Scheme 4:**
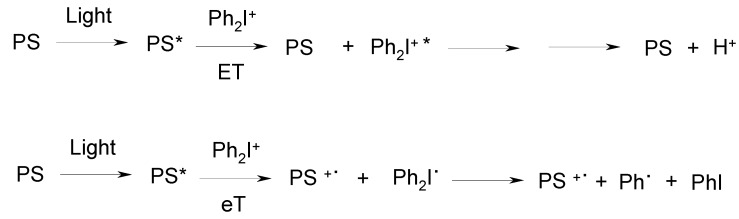
Energy (ET) or electron (eT) transfer to sensitize the decomposition of iodonium salts.

The photosensitized decomposition of Iod or Sulf in the presence of a suitable compound (denoted as a photosensitizer (PS) in photochemistry; in the RC area, PS can also be considered as a PI as it leads to initiating species) is possible. It occurs through energy (ET) or electron (eT) transfer processes governed by energetic considerations (in the usual triplet-triplet ET, the energy level of the excited donor must be higher than that of the acceptor) or thermodynamical requirements (the free energy change (ΔG) for the reaction must be negative), respectively (e.g., in Scheme 4). The possibilities for an ET route are rather limited (a low lying excited single state for getting a visible light absorption is not compatible with a high lying triplet state required for energy transfer).

On the opposite, the eT route is more successful in PS/Iod couples (this redox reaction is less favorable in PS/Sulf couples). Using, e.g., hydrocarbons, phenothiazines, ketones, dyes, metal complexes, various aromatic moiety linked to an epoxide unit, thiophene derivatives, poly(phenylene vinylene)s, as PS, a radical cation is produced (PS^•+^) and the formed diphenyl iodide radical cleaves into iodobenzene and a phenyl radical. The main drawbacks are the ability of PS^•+^ to initiate ROP (subsequent reactions of PS^•+^ with water, the phenyl radical or its dimerization have been shown in some cases) as long wavelength excitations and efficient PS/Iod electron transfer processes can be relatively easily obtained.

**Scheme 5:**
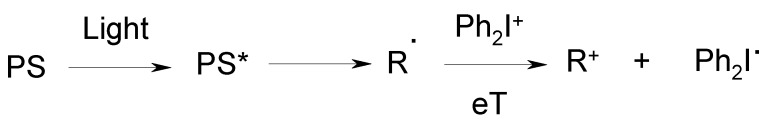
Oxidation of radicals to generate initiating species for ring opening polymerization (ROP).

The eT can also occur (Scheme 5) through an electron transfer between a radical R^•^ (generated from a cleavable PS) and Iod (the R^•^/Sulf redox reaction is very often not favorable due to the less favorable reduction potential of Sulf compared to Iod [5]). This way leads to a FRPCP reaction. Selecting PS allows changing the absorption but the initiating cation R^+^ is also changed (examples of such PS are ketones, ketone/amine, phosphine oxides, polysilanes, benzoyl germanes, dimanganese decacarbonyl/alkyl halide). The main drawbacks were the few available efficient systems (mostly restricted to the UV or near UV/vis range), the oxygen sensitivity of the system and the reactivity of R^+^. Another eT route concerns the addition/fragmentation process where R^•^ adds to a suitable allyl onium salt that further cleaves into a radical cation and an ethylenic product.

Recent successful results using PS/Iod systems and allowing visible light irradiations for ROP reactions can be found in, e.g., [43,44,45,46,47,48,49,50,51,52,53,54,55,56,57,58,59,60,61,62]. The design of one-component PI (e.g., the ferrocenium salts) is certainly an interesting answer to avoid such bi-component systems where compatibility and diffusion of the reactants in the polymerizable matrix might be a problem. In all cases current applications, however, except the use of Iod or Sulf alone under UV lights, a PS/Iod couple is conveniently employed as soon as UV radiations must be avoided. As stated above, in all these PS/Iod systems, the PS^•+^ initiating ability is decisive and the PS absorption has to match the emission spectrum of the light source: therefore, each couple will exhibit a characteristic behavior. Obviously, such systems cannot be versatile. A great progress has certainly been made using a novel strategy (see below).

Adequate structural modifications on Iod and Sulf led to compounds exhibiting red-shifted and enhanced absorption (e.g., bis triaryl sulfonium salts), suppressed benzene release (e.g., aryl iodonium salts) or allowed a better solubility (salts containing long alkyl chains) and compatibility (polymeric iodonium salts). Original and different cationic skeletons include dialkyl and cycloalkyl sulfonium salts, phenylethynyl sulfonium salts, acylsulfonium salts, dialkyl aryl sulfonium salts, thianthrenium salts or onium salts centered on a N (e.g., quinolinium, ammonium, anilinium salts, P (pyridinium, phosphonium salts), O (pyrilium salts) or a S atom (thiopyrilium, thiazolinium salts). Interesting results can be found in e.g., [63,64,65,66,67,68,69,70,71,72,73,74,75,76,77,78,79,80,81,82,83,84,85,86].

Other chemical structures that can initiate CP are e.g., the diazonium salts (e.g., in Ar_2_N^+^ BF_4_^−^, a BF_3_ Lewis acid is generated) [87,88]. Various organometallic derivatives (transition, non-transition and inorganic transition complexes) have also been proposed in the past [89] or recently revisited (see e.g., [70]); among them, the ferrocenium salt series (e.g., the (η^6^-cumene) (η^5^-cyclopentadienyl) iron (II) representative) ensure a blue light absorption: it works according to a ligand transfer reaction that expels the arene moiety, which is replaced by three epoxides, the initiating carbocation being obtained through a thermal cleavage of the epoxy C-O single bond (then, the polymerization proceeds outward through the coordination sphere of the iron atom). Non-ionic photoacid generators are also used (see e.g., in [90]).

The degree of separation in the propagating ion pair (oxonium cation/anion) is dependent on both the size and the electron density of the anion. Indeed, for this anion, with a larger size and/or a lower nucleophilicity, the polymerization rates become higher. Usual non-nucleophilic anions in onium salts allow excellent rates of polymerization, e.g., SbF_6_^−^ > AsF_6_^−^ > PF_6_^−^ > BF_4_^−^ >> I^−^ or Cl^−^. The bulky tetrakis (pentafluorophenyl) borate B(C_6_F_5_)_4_^−^ anion, which exhibits a low nucleophilicity together with a large size, leads to a high propagation rate constant and avoids Sb containing systems has ensured a substantial progress [91].

The UV intensity, temperature and dark-curing effects in CP/ROP have been recently considered again (see [92] and references therein).

The photoinitiated CP of renewable monomers (such as limonene 1,2-oxide and α-pinene oxide) was recently discussed ([93] and references therein).

## 3. Development of a New Strategy and Design of Novel Cationic Photoinitiating Systems

### 3.1. The New Strategy

**Scheme 6:**
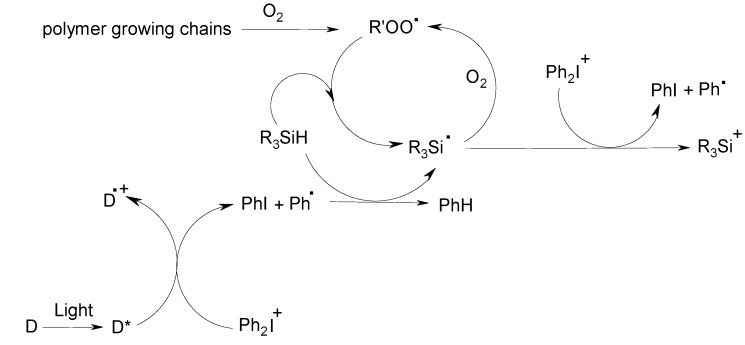
Photoinitiating systems using silyl radicals.

To avoid the drawbacks inherent to the photosensitization of cationic PIs in CP or the particular character of the FRPCP reactions observed so far (one kind of initiating radical cation PS^•+^ (Scheme 4) or cation R^+^ (Scheme 5) for one kind of PS in the PIS as recalled above), we have defined a novel strategy using the FRPCP procedure and being able to lead to the formation of the same initiating cation whatever the starting light absorbing compound (referred here as a dye D). This has been realized using a dye, a silane R_3_SiH such as tris-(trimethylsilyl)silane ((TMS)_3_SiH), or *N*-vinylcarbazole NVK) and an iodonium salt (such as Ph_2_I^+^, referred to as Iod) [94,95,96,97]. Upon light exposure, the excited dye (D*) (Scheme 6) reacts with Iod thereby generated a phenyl radical that interacts with R_3_SiH; the formed silyl radical R_3_Si^•^ is oxidized by Iod into a silylium cation R_3_Si^+^. Therefore, at any wavelength (this only depends on D), the same R_3_Si^+^ cation is produced. The criticism concerning a possible oxygen inhibition is ruled out as silyl radicals consume oxygen, scavenge all peroxyl radicals and regenerate new silyls (oxygen becomes a mediator in the initiating radical production). N-vinylcarbazole (NVK) appeared as a cheap and efficient alternative to the silane: the same mechanism is observed except that Ph^•^ adds to the NVK double bond (and formed Ph-NVK^•^) instead of abstracting a hydrogen atom on the silane. These three-component PISs (D/Iod/(TMS)_3_SiH or D/Iod/NVK) are really versatile and allows many possibilities as it will be seen below.

The decomposition of iodonium salts in the presence of a dye is largely encountered in the literature. The electron transfer reaction is usually fast as resulting from highly favorable free energy changes, but, unfortunately, back electron transfer reactions can more or less reduce the yield of electron transfer and decrease the overall efficiency of the system. The phenyl/silane hydrogen abstraction reaction is also a fast process. The silyl/iodonium salt interaction (contrary to that of silyl/sulfonium salt) is largely favorable. The silylium efficiently adds to the epoxide. Thus, the starting point of the research consists in looking for dyes D exhibiting suitable absorptions (from the UV to the red region) together with molar extinction coefficients ε as high as possible. In fact, the initiation step of a photoinduced CP is dependent on the amount of absorbed energy I_abs_ and the initiation quantum yield Φ_i_:I_abs_ is linked to the incident light intensity I_o_ of the light source and the ε of the dye (and obviously, path length l and reactant concentration c) according to I_abs_ = I_0_ (1 − 10^−εcl^) and the Φ_i_ term exhibits a rather complex dependence on the rate constants of the different excited state processes involved.

In addition to the use of already known dye structures [5], the development of highly absorbing dyes in the 380–700 nm wavelength range has required a search for novel skeletons, a specific design of novel architectures with a strong coupling of the molecular orbitals or the quest for compounds existing in other application areas.

Such dyes include (see a review in [27]): (i) modified known compounds such as ruthenium, iridium, iron and copper salts, titanocenes, benzophenones, thioxanthones, Michler’s ketones, thiopyrilium salts, metal carbonyls, decatungstates, polyoxometalates; (ii) commercial compounds such as violanthrone; (iii) available derivatives of structures in other areas such as thiophenes, dihydropyrenes or squarianes; and (iv) synthesized novel skeletons such as bi- and tri-functional compounds with an extending delocalization.

These dye/silane (or NVK)/iodonium salt combinations can easily work in very different experimental conditions. They all allow FRPCP under air. In the absence of silane or NVK, they initiate CP. Various aspects of such reactions as well as the characteristics of the PISs are the following.

### 3.2. Examples of What Can Be Achieved

**Figure 1:**
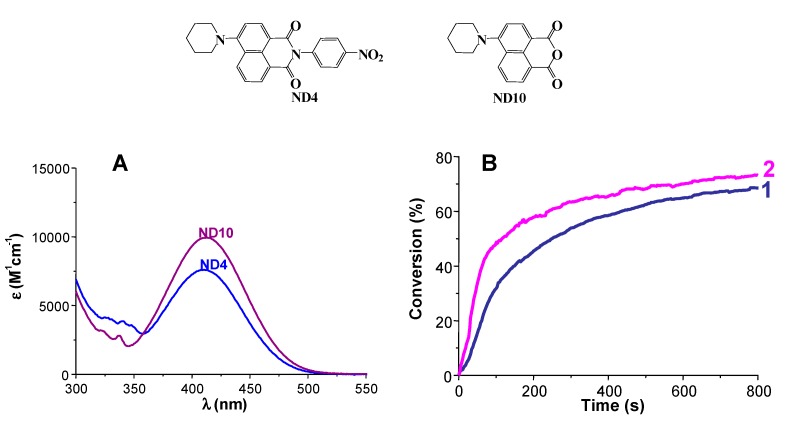
(**A**) UV-visible absorption spectra of ND10 and ND4; (**B**) Photopolymerization profiles for EPOX under air in the presence of (i) ND4/Iod/NVK (0.5%/2%/3%, *w*/*w*/*w*) upon the laser diode at 457 nm (curve 1) exposure; and (ii) ND10/Iod/NVK (0.5%/2%/3%, *w*/*w*/*w*) upon the laser diode at 457 nm exposure (curve 2) [105].

**Figure 2:**
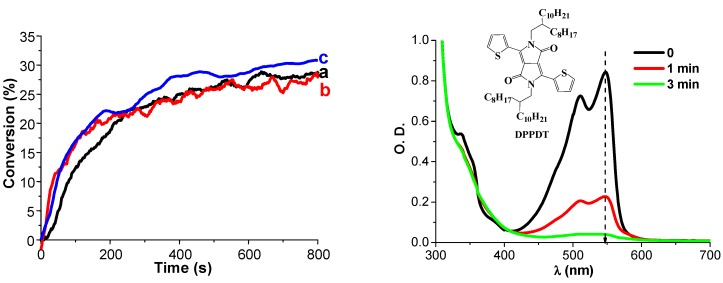
(left part) Photopolymerization profiles of EPOX under air in the presence of DPPDT/Iod (0.5%/2%, *w*/*w*) upon the halogen lamp (a) and laser diode at 532 nm (b); DPPDT/Iod/NVK (0.5%/2%/3%, *w*/*w*/*w*) upon the laser diode at 532 nm (c); (right part) Steady state photolysis of DPPDT/Iod in tetrahydrofuran upon the laser diode at 532 nm exposure under air, respectively; [Iod] = 3.1 × 10^–2^ M. from [110].

**Figure 3:**
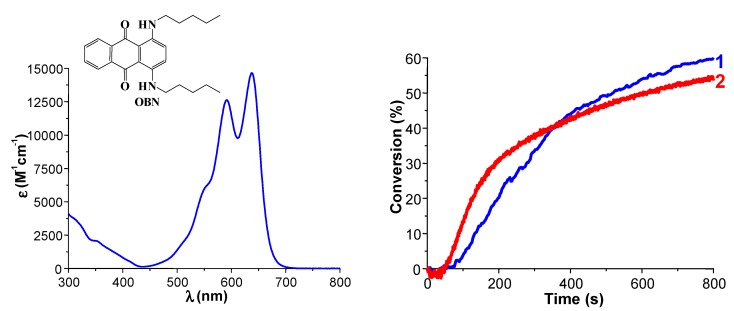
(**left**) UV-vis absorption spectrum for OBN and (**right**) Cationic photopolymerization upon red lights using an anthraquinone derivative (OBN) as photoinitiator: Photopolymerization profile of EPOX under air in the presence of (1) OBN/Iod/NVK (0.5%/2%/3%, *w*/*w*/*w*) upon the halogen lamp exposure; and (2) OBN/Iod/NVK (0.5%/2%/3%, *w*/*w*/*w*) upon the laser diode at 635 nm exposure [114].

***Use of Polychromatic lights.*** Polychromatic as well as monochromatic lights can be used. They range from the UV to the red. Possible sources are Hg, Xe-Hg or Xe lamps (band emission spectra or continuous spectra), laser diodes and LEDs but also low intensity household lamps (e.g., halogen lamps, fluorescent bulbs). With the best PISs, the conversions of (3,4-epoxycyclohexane)methyl 3,4-epoxycyclohexylcarboxylate (EPOX) used as a benchmark cyclic monomer for ROP are above 60% and can even reached 80%–90% under air upon exposure to various visible light sources having light intensities of 50–100 mW/cm^−2^. Competitive-reference PISs are ketone/Iod in the UV, phosphine oxides/Iod in the violet range, camphorquinone/Iod (optionally with alcohol) in the blue region, xanthene dye/Iod in the green range or polymethine/Iod in the red region. None of these systems is better than the best dye/Iod/silane (or NVK) combinations gathered in [27] for the ROP of 25 µm EPOX films in contact with air under exposure to relatively low light intensity sources. Many details can be found in a recent review [27]. Nice recent and particular examples (Figure 1, Figure 2 and Figure 3) include the ROP reactions of EPOX under air in the presence of suitable PISs upon exposure to various LEDs or laser diodes (80–100 mW/cm^2^):

- violet LED: dyes are, e.g., boranyls [98], benzophenones, thioxanthones, or benzoin ethers, phenylene, moiety coupled scaffolds [99,100,101,102]
- blue LED: dyes are, e.g., indanediones [103], pyrromethenes [104], naphtalimides [105], Ru [106] or Ir [107], complexes (Figure 1)
- green LED: dyes are, e.g., Ru [108] or Ir [109], complexes, diketopyrrolopyrroles [110] (Figure 2)
- red LED: dyes are, e.g., pentacenes [111], violanthrones [112], perylenes [113], anthraquinones [114] (Figure 3)

**Figure 4:**
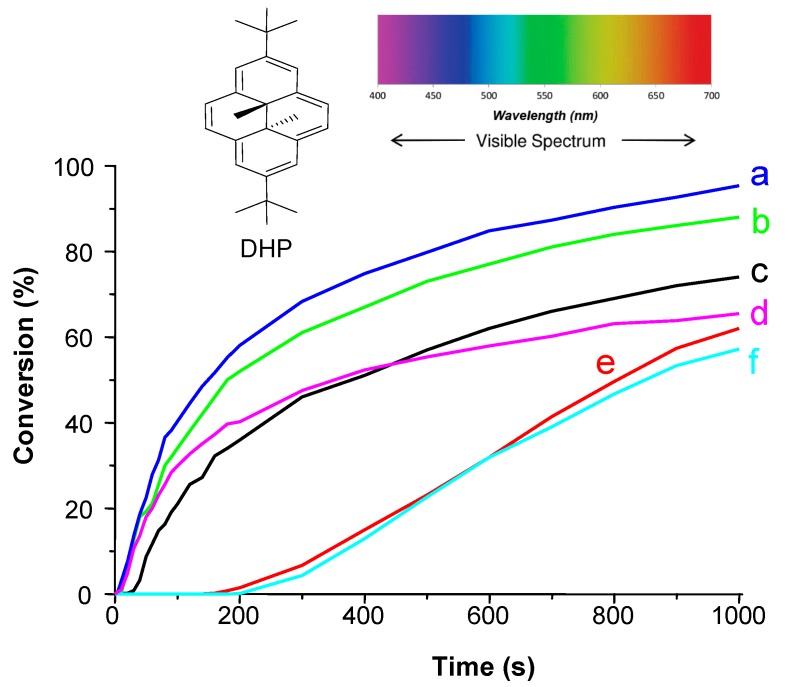
A multicolor photoinitiator for cationic polymerization and interpenetrated polymer network synthesis: 2,7-Di- *tert—*butyldimethyldihydropyrene (DHP) [115]: Photopolymerization profiles of EPOX under air using the dihydropyrene **DHP**/Iod/NVK photoinitiating system (1%/2%/3% *w*/*w*) upon irradiation with: (a) a laser diode (473 nm); (b) a laser diode (457 nm); (c) a halogen lamp; (d) a laser diode (635 nm); (e) a laser diode (405 nm); and (f) a laser diode (532 nm).

**Figure 5:**
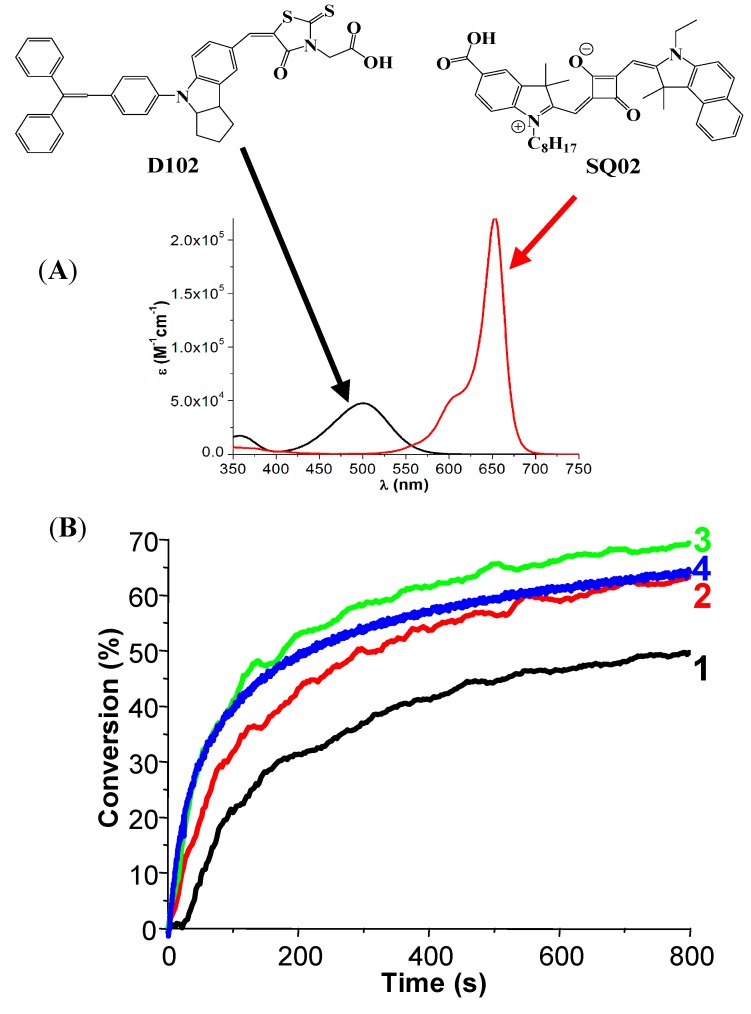
Panchromatic photopolymerizable cationic films using indoline and squaraine dye based photoinitiating systems [116]: (**A**) absorption spectra for the dyes and (**B**) Photopolymerization profile of EPOX under air in the presence of (1) D102/Iod (0.5%/2%, *w*/*w*) upon the halogen lamp exposure; and D102/Iod/NVK (0.5%/2%/3%, *w*/*w*/*w*) under the (2) halogen lamp; (3) laser diode at 457 nm; (4) laser diode at 473 nm. For mixture of photoinitiators (D102/SQ02), polymerization profiles are given in [116].

***Panchromatic PIs.*** The design of multicolour PI and panchromatic cationic formulations can also be achieved using a broad absorption band possessing dye (e.g., dihydropyrenes [115]) (Figure 4) or a careful mixing of several well adapted dyes, such as indanediones [103] and squaraines [116] (Figure 5).

**Figure 6:**
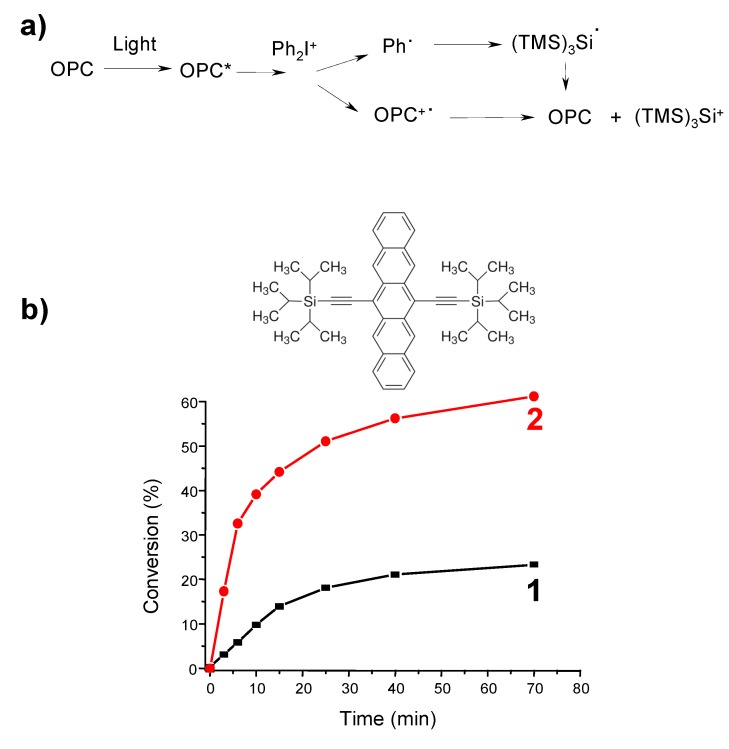
Tunable organophotocatalysts for polymerization reactions under visible lights: (**a**) reaction scheme for OPC and (**b**) polymerization profiles of EPOX under air upon: a red LED bulb irradiation in the presence of: (1) **Pent**/Ph_2_I^+^ (0.5%/2% *w*/*w*); and (2) **Pent**/(TMS)_3_Si-H/Ph_2_I^+^ (0.5%/3%/2% *w*/*w*) [111].

***Photoinitiator Catalysts.*** The elaboration of photoinitiator catalysts PIC was also described. In that case, the starting photoinitiator (dye) is recovered (and thereby not consumed) during the polymerization thanks to the use of adequate dyes as metal-based photocatalysts (MPC, e.g., [107,108,109]) or metal-free photocatalysts (organic photocatalysts OPC, e.g., pentacenes [111]) where redox reactions allow such behavior (Figure 6).

**Figure 7:**
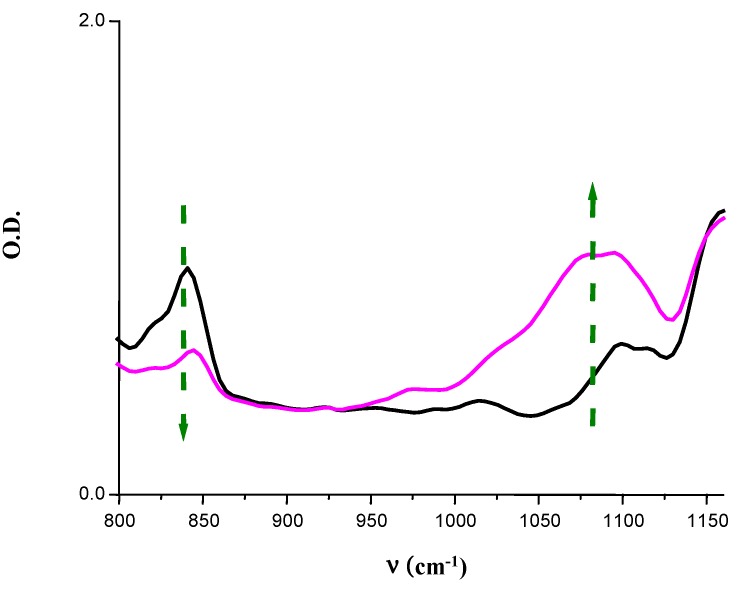
IR spectra recorded in the course of a photopolymerization of ELO; initiating system: BPSK/Ph_2_I^+^/TTMSS (1%/2%/3% w/w) upon a fluorescent bulb irradiation (from *t* = 0 to 30 min); under air [23].

***Renewable Monomers.*** The photopolymerization of renewable cationic monomers, that can be easily expected, has been demonstrated [117] (see also Figure 7). Tack-free coatings are formed. In any case, a decrease of the band at ~790 cm^−1^ (due to the epoxy ring) is monitored, whereas an increase of the IR absorption band of the polyether network is observed in the 1050–1150 cm^−1^ range.

***Polymerization of LDO, ELO and ESO.*** Due to the very excellent photosensitivity of the newly developed dye/silane (or NVK)/iodonium salt combinations, the irradiation under extremely soft irradiation conditions or sunlight exposure becomes feasible which should open new opportunities [23,117]. Figure 7 shows the epoxide consumption and the formation of the polyether network under a household fluorescent bulb (~10 mW/cm^−2^) or sunlight (<5 mW/cm^−2^) exposure under air. In outdoor conditions, tack-free coatings are obtained with LDO, ELO and ESO. As before, the polymerization profiles of these monomers are also clearly improved by the presence of a silane, e.g., for ELO, a tack-free coating is obtained within only 9 min in the presence of a silane (TTMSS) *vs.* 50 min in the absence of the silane [23].

**Figure 8:**
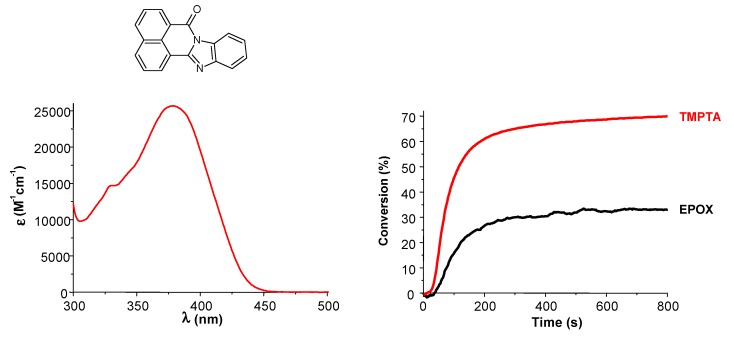
Interpenetrating polymer network production. Design of high performance photoinitiator (NA3) at 385–405 nm based on the naphthalene scaffold [118]: (**left**) absorption spectrum of NA3 and (**right**) Photopolymerization profiles of an EPOX/TMPTA blend (50%/50%, *w*/*w*) in the presence of NA3/Iod/NVK (0.5%/2%/3%, *w*/*w*/*w*) in laminate upon the halogen lamp exposure.

***IPN synthesis.*** As most of the proposed dye/silane (or NVK)/iodonium salt PISs are also able to generate radicals, the synthesis of interpenetrating polymer networks (IPN) through the concomitant photopolymerization of epoxide/acrylate blends (one-step hybrid cure) was efficiently carried out using suitable dyes, e.g., naphthalene, derivatives [118] (Figure 8).

**Figure 9:**
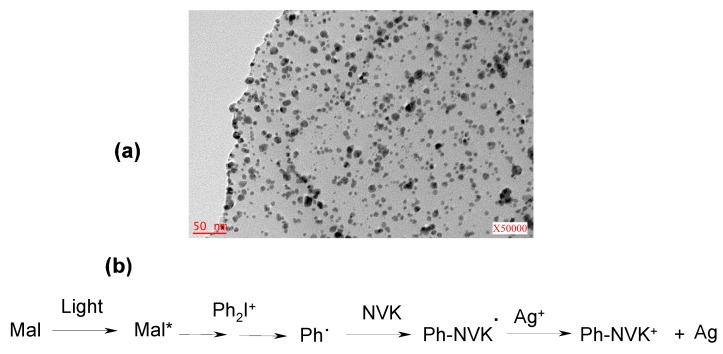
Push-pull malonate and malonitrile based dyes in photoinitiating systems for ROP and *in-situ* incorporation of silver nanoparticles in EPOX matrixes upon a Xe lamp exposure: (**a**) TEM micrograph and (**b**) chemical scheme for the formation of Ag(0)NP [119].

***Metal Nanoparticles.*** Finally, the simultaneous *in-situ* incorporation of metal nanoparticles (e.g., Ag) from a metal salt in the photopolymerizable cationic film using suitable PISs based on, e.g. malonates [119] was achieved (Figure 9). Other suitable reaction strategies have been very recently proposed: they allow the photochemical incorporation of nanoparticles NPs where the starting metal source does not exist as a salt: for example, Ti NPs have been generated in a photopolymerized EPOX matrix.

## 4. Conclusions

This paper shows that CP and FRPCP of cyclic monomers (here the ROP of a representative diepoxide) can now be easily triggered at any wavelengths, under air, even under low light intensity sources (LEDs, household lamps...), using more or less viscous monomer formulations, in the presence of novel photoinitiating systems. These recent results have likely introduced a breakthrough in the development of cationic PISs under visible lights, which was relatively restricted so far (although some interesting and successful results have been obtained with the conventional PISs as recalled above). Up to now, 400–700 nm light induced polymerization reactions were conveniently achieved mostly when using radical monomers. Therefore, although they present some drawbacks (e.g., oxygen inhibition, shrinkage), radical matrices were required in many applications aiming at employing soft irradiation conditions, laser lines or LEDs. According to the degree of performance attained, it seems today that cationic matrices can be used with the same success in these experimental conditions together with decisive arguments (e.g., no oxygen inhibition and less shrinkage). This certainly opens a lot of promising novel applications in various areas.
